# Artificial Teeth on the Atmospheric Principle

**Published:** 1851-01

**Authors:** W. A. Palmer

**Affiliations:** Po'keepsie


### ARTICLE XIV.

### *Artificial Teeth on the Atmostpheric Principle.*

MR. EDITOR: In the Recorder for December, one or two articles upon the above topic occur, which induces me to offer the following, in regard to my own experience in this matter, and if you see fit to give it a place in your periodical, you are at liberty to do so.

Having employed, in my own practice, for a length of time past, a similar plan to the one alluded to by yourself and correspondent, (the same in principle,) and with such results as lead me to hope much, ultimately, from the utility of the thing. As the processes I employ to attain it, are, I think, more simple than either of those described in the Recorder, I will here give them, and I doubt not you will agree with me, that an invention of this nature, is likely to be made useful in its practical bearing, in proportion as we can simplify the processes through which the operation has to go, to accomplish the end proposed.

My method is as follows:—When I have taken a correct impression of the mouth in wax, and obtained from it the plaster model, I take a mixture of prepared chalk, gum arabic and water, of the consistency of cream, and with the aid of a camel's-hair pencil, build upon that part of the plaster cast, representing the alveolar ridge, a prominence, say one-twelfth of an inch in width, of a conical form, and about the thickness of an American half dollar, to extend as far back as the first bicuspid on either side, in a perfect half circle, diminishing in thickness as it approaches its terminus, until lost in an even surface on the cast. This crescent or half circle being allowed to incline a little inward toward the point where the linings of the teeth would naturally terminate, or be joined, by soldering, upon the plate, and being partly covered as it would be by the base of each tooth on that part of the plate ; it may reaidly be seen that but little, if any, objection could be urged, on the score of making the plate more cumbersome in the mouth. In fact, it is susceptible of being so arranged that this additional vacuum in the plate can scarcely be noticed by the wearer. A plaster model thus formed, enables the operator, at once, without the need of resorting to the old and tedious methods of using founder's sand, to make good and correct metal casts, as follows: Take a common iron ladle, melt five or six pounds of lead, then take the plaster model after all moisture is removed therefrom, and hold it in the lead until it is perfectly congealed, then, after allowing the metal time to cool, so as to handle, break out the plaster, and the hollow cast is made with which to mould the convex cast. After cleansing out the hollow cast, and giving the inner surface a coating of chalk, then place a narrow rim of sheet iron around the margin of the hollow cast, securing the outer edges with a mixture of plaster and sand; then pour a sufficient quantity of melted tin into this mould to fill it and the rim around it, and the two casts are made.

I have been thus minute, because I am inclined to think much time is wasted by many dentists, in using means for getting up casts, which are far more complicated, and which are not found to be any better in the result after all. In some cases where the mouth of a person presents, upon the alveolar ridge, an uneven surface, I have built up only where the depression occurred, thus elevating the one to the level of the other, thereby giving more symmetry and beauty to the case when finished, and still having the adhesive qualities of the plate sufficiently increased to answer a good purpose. In several cases of this nature, I have been well pleased with the result.

Other chambers or vacuums could, I am persuaded, be made to advantage in a plate, in addition to the one already described. For instance, say two separate ones—one on each side of the plate, toward the posterior part of the alveolar ridge of the length of half an inch—commencing at a point about the center of the second molaris, and running in a transverse direction toward that point where the plate turns over the alveolar process.

This would doubtless increase the adhesion of the plate at its posterior base, and would, I doubt not, be worth the trial. This last, however, I have not made trial of.

With regard to the question of *priority* in the employment of this plan of using the atmospheric principle, I must conclude that we shall have to go farther back than we have yet been doing, in order to place the credit of *its discovery* where it belongs.

The gentleman in your city, to whom I am candid to say I take pride in acknowledging myself indebted for the first sugtion of the idea, is able to show, by an entry in his register, a case inserted in the mouth of a gentleman, as long ago as 1836, on this plan, and I am *not aware* that even he lays claim to the first discovery of the thing. Liberal minds are hardly likely to lay much stress on points like this, much less to be found *taking out letters patent* for discoveries in matters pertaining to a profession like ours.

Po'keepsie, *Jan'y* 10, 1848. W. A. PALMER.

[*Dent. Rec.*

